# A Literature Review of Underlying Molecular Factors Contributing to the Pathogenesis of Diabetic Eye Disease

**DOI:** 10.7759/cureus.106364

**Published:** 2026-04-03

**Authors:** Neelay Inamdar, Monika Deshpande

**Affiliations:** 1 Department of Medicine, Rowan-Virtua School of Osteopathic Medicine, Rowan University, Stratford, USA; 2 Department of Ophthalmology, Wilmer Eye Institute, Johns Hopkins University School of Medicine, Baltimore, USA

**Keywords:** diabetic eye disease, diabetic retinopathy, epo, fibroblast growth factor (fgf), hypo-glycemic, hypoxia-inducible factor (hif)-1α, metabolic changes and diabetes, vegf angiogenesis

## Abstract

Diabetic eye disease, the leading microvascular complication of diabetes mellitus (DM), is one of the leading causes of blindness worldwide, whose disease burden and demographics are only expected to grow in the coming decade. Advances in molecular biology techniques have enabled the identification and study of several proteins and transcription factors believed to play key roles in the underlying disease pathogenesis. A majority of these factors work together, contributing to both angiogenic (formation of new blood vessels) and inflammatory processes underlying diabetic retinopathy (DR).As a result, emerging therapies are increasingly targeting specific molecular mechanisms. In parallel, nonpharmacological interventions are being proposed to inform the development of appropriate clinical diagnostic and treatment guidelines. These approaches aim to address the early stages of disease and slow or prevent progression to chronic, later stages that may result in vision loss. This review synthesizes foundational and recent evidence using a qualitative narrative approach, focusing on hypoxia-driven molecular pathways rather than quantitative meta-analysis. Although numerous studies have consistently identified key molecular factors that contribute to the pathogenesis of diabetic eye disease, additional details regarding the specific roles of some factors listed herein, as well as the discovery and involvement of other factors in the pathway, remain to be fully explored and understood.

## Introduction and background

Diabetic eye disease, specifically, diabetic retinopathy (DR), is the most common microvascular complication of diabetes mellitus (DM) and remains the leading cause of blindness in working-age individuals in most developed nations worldwide [1,2]. Clinically, DR exists along a well-defined spectrum, ranging from early non-proliferative diabetic retinopathy (NPDR), characterized by microaneurysms, intraretinal hemorrhages, and vascular leakage, to advanced proliferative diabetic retinopathy (PDR), marked by pathological retinal neovascularization. Diabetic macular edema (DME), which can occur at any stage, represents a major cause of vision loss due to fluid accumulation within the macula.

Since the invention of the ophthalmoscope in 1851, in vivo fundoscopy has provided a means of identifying and visualizing the manifestations of changes to the retina in response to various diseases. However, it was not until 1872 that the first histological confirmation of diabetic changes in the retina was documented in patients with DM [3]. In the years since, advancements in laboratory techniques such as enzyme-linked immunosorbent assay (ELISA), immunohistochemistry, and polymerase chain reaction, along with imaging modalities such as fluorescein angiography, have allowed researchers to investigate the underlying pathogenesis of DR more deeply. These tools have facilitated efforts to better understand the relationship between the chronic morbidity of DM and the clinical outcome of vision loss, with the goal of developing therapeutic strategies targeting key molecular mediators involved in disease progression [3].

A hallmark central pathological trigger in DR is retinal hypoxia, which activates angiogenic and inflammatory cascades and leads to pathological retinal neovascularization. These newly formed blood vessels are deficient in tight junctions, making them prone to leakage, rupture, or collapse [4]. These changes can result in vitreous hemorrhage or tractional retinal detachment, either of which can significantly impair the signal transduction pathways responsible for clear vision, ultimately leading to permanent vision loss [4]. Hyperglycemia, the defining feature of DM, is a major contributor to the hypoxic conditions that predispose the retina to both structural and physiological complications. This metabolic stress promotes the production of angiogenic, inflammatory, and oxidative stress-related molecular mediators in an attempt to combat the metabolic insult and restore tissue oxygen homeostasis [5]. At the molecular level, DR is driven by interconnected pathways including hypoxia-inducible factor-1α (HIF-1α) signaling, vascular endothelial growth factor (VEGF)-mediated angiogenesis, inflammatory cytokines, oxidative stress responses, and metabolic regulators, which together shape disease progression. Over time, sustained activation of these pathways leads to maladaptive vascular remodeling, chronic inflammation, and progressive retinal dysfunction.

Importantly, as DR is a progressive process caused by long-term functional impairment of the retina, early disease is frequently asymptomatic. By the time the symptoms of vision loss begin to set in, in most cases, irreversible damage to the retina may already have occurred. This underscores the need for mechanistic understanding and early intervention [5]. Current treatment strategies for PDR involve angiogenic inhibitors, anti-inflammatory agents, and antioxidants with increasing emphasis on early detection and mechanism-based preventive intervention to slow disease progression [5,6]. Despite these pharmacological advances, traditional interventions like retinal laser therapy, specifically panretinal photocoagulation, remain a cornerstone in the management of PDR and continue to play an essential role in the current clinical toolkit. As mentioned before, DR is now recognized as a multifactorial disease driven by interconnected signaling networks rather than a single dominant pathway. Central molecular themes discussed in this review include HIF-1α signaling, VEGF-mediated angiogenesis, inflammatory cytokine activation, oxidative stress responses, and metabolic regulators that together shape the retinal microenvironment.

This literature review aims to synthesize the results of experimental studies and review articles from the past 40 years to create a cohesive overview of the molecular mechanisms widely believed to play a role in the complex, multifactorial pathogenesis of DR and diabetes-related vision loss.

## Review

Methods

Review Design

This article is a narrative literature review intended to synthesize key mechanistic insights into the molecular pathogenesis of DR rather than a systematic review. As such, formal risk-of-bias assessment, quantitative syntheses, and meta-analytic procedures were not performed. Consistent with this qualitative approach, the methodology was designed to support mechanistic interpretation and conceptual integration rather than an exhaustive systematic identification of all available studies.

Information Sources

We conducted a comprehensive review of both recent and historical literature using PubMed, Embase, SCOPUS, and Google Scholar. Searches were performed independently across databases without formal database-specific protocol standardization or harmonization of search syntax.

Search Strategy

A consistent set of search terms was applied across databases, including: “Diabetes AND Eye Disease,” “Diabetic Retinopathy,” “HIF-1α,” “Diabetes AND HIF-1α,” “ANGPTL-4,” “VEGF,” “IGF-1,” “FGF,” “EPO,” “Hypoxia,” and related combinations. Searches were conducted independently across databases, and duplicate records were removed manually prior to screening. Formal reproducibility parameters such as predefined Boolean logic strings, database-specific query adaptations, and/or standardized search documentation were not prospectively specified. Accordingly, the search strategy reflects an iterative, investigator-driven approach rather than a fully reproducible systematic protocol.

Study Selection and Inclusion Criteria

Studies published in the English language between 1985 and 2024 were considered. Primary research articles, review articles, systematic reviews, and meta-analyses relevant to the molecular mechanisms of diabetic eye disease were included. Full-text articles were reviewed when abstracts indicated relevance to hypoxia signaling, angiogenesis, inflammation, metabolic regulation, or therapeutic implications. Titles and abstracts were screened for relevance to the molecular and pathophysiological mechanisms of DR. Studies lacking mechanistic relevance to retinal pathology were excluded. No restrictions were imposed on the study population or clinical outcome measures, provided the study contributed mechanistic insight into diabetic retinal disease. Screening was conducted without a predefined, trackable workflow (e.g., no formal accounting of records identified, screened, or included), and a PRISMA-style flow diagram was not generated. Inclusion criteria were intentionally broad to capture diverse mechanistic evidence. No explicit hierarchy of evidence (e.g., prioritization of human clinical data over preclinical models) was applied, which may introduce variability in evidentiary weighting and potential selection bias.

Data Synthesis

A structured qualitative narrative synthesis was performed. Conflicting evidence was interpreted in the context of study design, experimental model, and disease stage. No formal statistical summary methods (e.g., meta-analysis, meta-regression, or pooled quantitative synthesis) were planned or performed. Given the heterogeneity of the included literature, including in vitro studies, animal models, biomarker analyses, and clinical observational data, a qualitative narrative synthesis approach was employed. This strategy was chosen to integrate mechanistic insights across diverse study designs, outcomes, and populations that are not amenable to statistical pooling. Findings were qualitatively synthesized to identify recurring molecular pathways, signaling hierarchies, and mechanistic patterns contributing to the development and progression of DR. Data were synthesized using a structured qualitative narrative approach, prioritizing consistency of mechanistic findings across experimental, translational, and clinical studies.

However, no predefined conceptual framework was used to systematically categorize pathways (e.g., hypoxia-driven, inflammatory, or metabolic), and organization of mechanisms was performed thematically based on narrative synthesis. This may result in a more descriptive rather than fully hierarchical or integrative presentation.

Similarly, discordant findings were considered in context. For instance, some modulatory and context-dependent factors demonstrated paradoxical roles in this review. However, no formal weighting schema or standardized criteria were used to reconcile conflicting evidence or prioritize specific molecular pathways. Instead, interpretive emphasis was placed on consistency across studies and biological plausibility.

Future refinement of this approach can possibly include adoption of a predefined mechanistic framework, structured pathway classification, and incorporation of visual synthesis tools (e.g., summary tables or schematic pathway diagrams) to enhance methodological transparency, reproducibility, and translational clarity.

Review of molecular mechanisms

Throughout the review, molecular pathways are discussed using converging evidence from experimental, translational, and clinical studies. Findings derived primarily from preclinical models are explicitly noted to distinguish mechanistic inference from direct clinical evidence. In contrast, associations supported by human studies are identified as such to provide appropriate context regarding the strength and consistency of the evidence.

HIF-1α Is an Upstream Regulatory Hub That Functions as a Master Regulator of Cellular Responses to Retinal Hypoxia

HIF-1α: Several studies have identified HIF-1α as a key contributor to the pathogenesis of DR. HIFs are transcription factors that serve as major regulators of the cellular adaptive response to hypoxia. These proteins can simultaneously activate hundreds of target genes to reverse hypoxia and restore and maintain cellular oxygen homeostasis in various tissues, including the retina [7]. Among the multiple HIF isoforms and subtypes, HIF-1 is induced earliest in response to hypoxia, and HIF-1α has emerged as the most ubiquitously expressed and extensively studied subtype [7]. Under normoxic conditions, HIF-1α has an extremely short half-life, as its intracellular levels are strictly regulated by the proteasomal degradation pathway. However, under hypoxic conditions, it evades degradation and translocates to the nucleus, where it exerts its gene expression effect and activates multiple other pathways that can contribute to both adaptive and pathological mechanisms of DR [8].

Several inflammatory cytokines, including interleukin-6 (IL-6)-6 and tumor necrosis factor-alpha (TNF-α), have been implicated in this process, with their levels elevated in response to retinal ischemia [9]. Interestingly, DM alters neovascularization in a context-dependent manner, promoting angiogenesis in the retina while inhibiting vascular formation in the peripheral nervous system. The effects of HIF-1α in the tissues are also similarly dependent on other contextual factors, including the duration of hypoxia; while acute hypoxia may activate protective responses, chronic hypoxia may promote oxidative stress, inflammation, and cell death [10].

While hyperglycemia has been continuously implicated in the link between HIF-1α and DR, emerging evidence indicates that the hypoglycemic state may also paradoxically elevate the expression of HIF-1α and contribute to DR progression [10]. Mechanistically, this may occur due to the HIF-1-dependent increased expression of the GLUT1 transporter protein in retinal Muller glial cells in response to hypoglycemia, which consequently increases expression of the angiogenic mediators under hypoglycemic conditions [11]. This paradox underscores the complex regulatory role of HIF-1α in retinal homeostasis and disease.

Although HIF-1α plays a role in the molecular pathology of DR, it does not work in isolation. Rather, its activation initiates a cascade of downstream signaling molecules and molecular factors, ultimately leading to the progressive vascular and neuronal damage characteristic of DR, and eventual vision loss.

Downstream Angiogenic Effectors: VEGF and Angiopoietin-Like Protein 4 (ANGPTL-4) Mediate Endothelial Proliferation, Vascular Permeability, and Inflammatory Signaling in DR

VEGF: VEGF is one of the major downstream effectors of HIF-1α. A pivotal study supporting this relationship demonstrated that inhibition of HIF-1α suppressed VEGF expression and deferred the retinal angiogenesis in a time-dependent manner [12]. The biological function of VEGF involves regulation of physiological angiogenesis during embryonic development as well as wound healing, and also in pathological conditions such as tumor growth and neovascular and proliferative disorders [13].

VEGF meets all the required conditions for promoting angiogenesis, such as increasing vascular permeability and endothelial cell migration, proliferation, and tube formation. It has two major high-affinity receptors with cytoplasmic tyrosine kinase domains, VEGFR-1 and VEGFR-2, both of which are expressed on endothelial cells, including those in the retina. Under hypoxic conditions, endothelial cells have increased expression of VEGF, suggesting that this increase may be due to increased transcriptional activation of these genes in response to the metabolic stress of hypoxia, much like other hypoxia-sensitive factors such as erythropoietin (EPO) [14]. The PI3K/Akt pathway induces VEGF-A, the most potent VEGF isoform, at both the mRNA and protein levels in streptozotocin-induced diabetic mice, a model that recapitulates key molecular features observed in human DR. The signal transduction pathway mediating this is believed to be regulated by the epidermal growth factor-like structural domain 7 (EGFL7) protein in retinal endothelial cells, although the exact mechanism and a definitive link to the pathogenesis of DR have not yet been fully determined [15].

Collectively, these studies support the central role of VEGF as a major pro-angiogenic mediator, not only in normal physiological responses to hypoxia and tissue remodeling, but also in the pathological neovascularization characteristic of DR. Its regulation by HIF-1α and other hypoxia-driven pathways underscores VEGF’s significance as a therapeutic target in preventing or mitigating vision loss in diabetic patients.

ANGPTL-4: In addition to VEGF, the protein ANGPTL-4 has emerged as another key contributor to the pathological retinal neovascularization observed in diabetic conditions. ANGPTL-4 is a glycoprotein involved in cardiovascular and retinal diseases and works as a regulator of glucose and lipid metabolism. It regulates triacylglycerol homeostasis in fasting conditions and stimulates intracellular lipolysis. It also saves the body from excess triglyceride hydrolysis and stimulation, thereby acting as a homeostatic modulator as opposed to a unidirectional effector in the body’s own mechanisms of counteracting environmental or metabolic stress responses [16].

High glucose levels increase the expression of ANGPTL-4 in combination with HIF-1α. This highlights the fact that HIF-1α is not the sole mediator of retinal microvascular proliferation. Rather, it functions in concert with downstream effectors such as ANGPTL-4. Both in vitro and in vivo studies have demonstrated that ANGPTL-4 promotes diabetic retinal inflammation and angiogenesis through profillin-1, an actin-binding protein involved in the regulation of the inflammatory response. Since DR is now increasingly recognized as a condition driven by both angiogenesis and inflammation, ANGPTL-4’s dual contribution supports its role in the pathogenesis of DR.

ANGPTL-4 has systemic metabolic effects: it improves glucose tolerance and induces hyperlipidemia and hepatic steatosis, which suggests that, in combination with hyperglycemia, it also potentially influences the dyslipidemia associated with type 2 diabetes mellitus (T2DM) [17]. In addition to HIF-1α, ANGPTL-4 also acts synergistically with VEGF, the other major downstream regulator of vascular proliferation in DR, whose levels also increase in response to HIF-1α. ELISA analyses revealed significantly elevated vitreous levels of both VEGF and ANGPTL-4 in patients with PDR compared to nondiabetic patients with ocular diseases such as idiopathic macular hole (IMH). Additionally, the vitreous and serum levels of ANGPTL-4 also correlated with serum triglycerides and high-density lipoprotein (HDL) levels, further suggesting its multifaceted roles in DR [18].

While ANGPTL-4 clearly plays a role in the pathogenesis of DR, both independently and in conjunction with other molecular pathways, its precise mechanistic contributions remain incompletely understood. Further elucidation of its signaling networks could reveal new therapeutic targets for addressing the complex processes of inflammation, neovascularization, and metabolic dysfunction in diabetic eye disease.

Modulatory and Context-Dependent Factors: Insulin-Like Growth Factor 1 (IGF-1), Fibroblast Growth Factor (FGF), and EPO Act As Mediators That Influence Angiogenesis and Inflammation in a Context-Dependent Manner, Reflecting Metabolic and Hypoxic Conditions

IGF-1 and FGF: While VEGF and ANGPTL-4 function downstream of HIF-1α in the partially understood mechanism of PDR, additional modulatory and context-dependent molecular mediators have been implicated in the angiogenic and inflammatory processes that characterize the disease. One such factor is IGF-1. IGF-1 is a chemical messenger hormone that mediates the effects of growth hormone (GH) on the body, contributing to the normal growth of bones, tissues, and organs. Unlike those of GH, which exhibit diurnal and activity-dependent fluctuations, IGF-1 levels remain relatively stable, making it a more reliable diagnostic biomarker in conditions such as acromegaly and achondroplasia. In an experimental study by Grant et al., exogenous IGF-1 administration into the normally avascular environment of the rabbit cornea induced angiogenesis, implicating IGF-1 in vascular proliferation [19]. Notably, these effects were comparable to those observed with FGF, another protein involved in tissue repair, regeneration, and angiogenesis.

Beyond their individual contributions, both IGF-1 and FGF interact synergistically with VEGF, enhancing its expression and promoting the proliferation of vascular endothelial cells, which are essential for the formation and maintenance of both macro- and microvasculature. Neurotrophic factors in the retina stimulate the retinal Muller cells to produce FGF, which then activates VEGF, which contributes to the production of endothelial cells. Given that FGF receptors are expressed in the retina, this supports a role for FGF in the formation of epithelial membranes. This implicates the FGF, alongside IGF-1, in the pathogenesis of DR. These findings underscore the interdependence of growth factor signaling, angiogenesis, and metabolic demand in retinal tissue development and disease.

As growing evidence points to the process of DR as being inflammatory in nature, many of these growth factors, particularly IGF-1 and FGF, could potentially serve as biomarkers of inflammation, contributing to the angiogenic signaling. Their interactions with VEGF, and potentially with HIF-1α, suggest a complex and coordinated regulatory network. Although the link between IGF-1, FGF, and VEGF in DR is well supported, the precise molecular mechanisms connecting IGF-1 to HIF-1α remain incompletely understood and warrant further investigation [20].

EPO: Although VEGF, ANGPTL-4, and IGF-1 are recognized as key contributors to the process of DR, the role of EPO has garnered increasing attention. While EPO has long been known to be produced in the kidney and is known for its role in erythropoiesis in response to hypoxia, emerging evidence suggests that it also plays broader cryoprotective and tissue-reparative roles, including in the retina. In response to tissue injury, EPO appears to be released as a protective agent, potentially mediated by the activity of the tissue protective receptor (TPR). Importantly, studies using diabetic rat models have shown that EPO may downregulate HIF-1α expression and suppress VEGF expression, indicating that it may attenuate, rather than promote, the inflammatory and angiogenic response associated with DR, at least in the early stages of the disease. These findings suggest that although EPO is induced by hypoxia, similar to VEGF and ANGPTL-4, it may initially function in a paradoxical modulatory or counter-regulatory role rather than directly exacerbating neovascularization.

Preliminary clinical investigations have explored the therapeutic potential of exogenous EPO in DME and early stages of DR, with some studies reporting beneficial outcomes. However, the safety profile of EPO remains controversial, especially given concerns about its angiogenic potential, possibly in the later stages, and the lack of clarity surrounding its mechanistic role in DR. As such, further research is needed to elucidate whether EPO exerts a net protective or pathogenic effect in diabetic retinal disease [21].

Recent studies have attempted to explore the genetic basis of this variability, specifically the role of transcription factor-7-like-2 (TCF7L2), a gene involved in beta cell function and susceptibility to T2DM. Polymorphisms in TCF7L2, associated with a higher risk of developing T2DM, may influence the balance between protective and deleterious EPO activity. Specifically, one study demonstrated that EPO and TCF7L2 were in equilibrium in both DM and control conditions, suggesting a possible regulatory interaction [22]. Notably, certain EPO alleles, such as G and C, are associated with a net protective effect against DR, while A and T alleles confer a deleterious effect in DR. Although the exact mechanisms by which these polymorphisms influence DR progression remain unclear, data support a genotype-dependent role for EPO in retinal pathology. Additionally, elevated intraocular EPO concentrations have been observed in patients with proliferative DR, implicating EPO in disease progression.

Despite these insights, the extent to which TCF7L2 polymorphisms and EPO variants contribute to DR remains incompletely understood. Further studies are warranted to characterize these gene-protein interactions and determine their clinical relevance, particularly in diverse populations. While EPO gene polymorphisms may contribute to individual susceptibility to DR, their predictive utility and mechanistic relevance are still under investigation [22]. EPO is also associated with the regulation of caspase-3 and oxidative stress pathways, further reinforcing the hypothesis that DR results, in part, from a dysregulated stress response to hypoxia and hyperglycemia.

Discussion

The mechanistic pathways described in this review can be best understood within a hierarchical framework. The upstream hypoxia-driven signaling, primarily mediated by HIF-1α, initiates a cascade of downstream angiogenic and inflammatory responses. Key downstream effectors include VEGF and ANGPTL-4, which promote vascular permeability and neovascularization. Factors such as IGF-1, FGF, and EPO modulate these processes in a context-dependent manner. Framing these pathways within an upstream/downstream signaling axis highlights their interdependence and facilitates conceptual integration across molecular pathways.

It is important to consider how this molecular framework is reflected at the structural level within the retina. While retinal capillary non-perfusion is not a focus of this molecular review, this important pathophysiological component strengthens the link between microvascular changes and molecular signaling. It represents a critical structural endpoint in which the permanent closure of small blood vessels creates silent zones within the macular and peripheral zones. This clinical feature serves as a biologically relevant substrate, as these areas of stalled blood flow act as a catalyst for chronic local ischemia rather than just a passive sign of damage. Through physical alteration of the retinal architecture, non-perfusion directly interacts with hypoxia-responsive pathways, triggering a molecular cascade that drives the progression of proliferative diseases.

Taken together, these interconnected molecular and structural processes provide a broader context for understanding the downstream clinical manifestations of DR.

Diabetic Retinal Neurodegeneration and Inflammation

The pathophysiology of DR is multifactorial, and the condition arises from the cumulative, chronic effects of untreated or poorly managed DM. The extraocular factors most associated with the risk and progression of DR include poor glycemic control, hypertension (HTN), dyslipidemia, and genetic predisposition. These combined states appear to drive the body’s mechanistic responses aimed at restoring homeostasis in the face of prolonged metabolic stress. Studies involving HIF-1α suggest that, in response to ischemia, the body activates this factor to stimulate microvascular proliferation, thereby attempting to correct the underlying pathology. HIF-1α acts in conjunction with a number of other angiogenic mediators such as VEGF, ANGPTL-4, IGF-1/FGF, and the more recently implicated EPO, all of which contribute to the progression of DR. As understanding of these molecular pathways has expanded, so too has the effort to categorize the clinical manifestations of DR into standardized classification systems. These systems stratify patients by disease severity, such as mild to moderate proliferative DR or mild to severe macular edema, enabling tailored diagnosis and management. Current treatment options include vitrectomy, laser photocoagulation, and increasingly, pharmacotherapy with anti-VEGF inhibitors, which target some of the major angiogenic pathways [23].

*Therapeutic Implications and Future Directions* While anti-VEGF therapies remain central to DR management, important limitations persist, including incomplete reversal of ischemia and variable long-term durability. There is limited development of drugs targeting alternative pathophysiologic pathways. Newer agents such as faricimab and multi-pathway approaches highlight the need for broader molecular targeting strategies. Faricimab is a bi-specific monoclonal antibody that targets both VEGF and the angiopoietin-Tie2 pathways. This dual-action mechanism is thought to result in greater vascular stability and reduced leakage, offering synergistic benefits. As new molecular insights emerge, it is increasingly clear that the current classification system for DR requires revision, not only to incorporate newly discovered vascular factors but also to address growing interest in the non-vascular components of DR, such as diabetic retinal neurodegeneration and diabetic retinal neural dysfunction. This idea has gained traction due to advances in structural retinal imaging and functional assessments.

While these therapies have demonstrated some benefit, their widespread efficacy remains to be determined, and their impact on other molecular pathways remains limited. Notably, DR is now recognized not only as an angiogenic, but also as an inflammatory condition. Inhibiting HIF-1α reduces inflammatory signaling, including downregulating the expression of IL-6 and TNF-alpha and their receptors. This points to the potential for early therapeutic intervention aimed at inflammation to prevent or slow the development of PDR under hypoxic conditions associated with DM.

Therefore, the focus has been on the regulation of the relative levels of HIF-1α rather than on the absolute levels of HIF-1α. Current strategies emphasize the regulation of its relative expression to achieve therapeutic balance while minimizing adverse effects.

The expanding knowledge base highlights the urgent need for updated classification schemes and diagnostic criteria to guide optimal treatment. As the global burden of DR continues to grow, so too does the need for a better understanding of the additional underlying molecular factors beyond VEGF, ANGPTL-4, and HIF-1α. These insights could facilitate development of new therapies that are safe, effective, and scalable for implementation in high- and low-resource settings [24]. Because DR involves multiple biological systems, its treatment requires a multidisciplinary approach. Interprofessional collaboration and strong therapeutic relationships between healthcare providers and patients are essential to effectively screen and develop individualized treatment plans for the management of DM, as well as ensure appropriate long-term follow-up.

Annual ophthalmological exams remain a cornerstone of DR screening. DR has been linked to other systemic complications of DM, such as stroke, myocardial infarction, diabetic nephropathy, and diabetic neuropathy. Although tight glycemic control remains the primary strategy for DM management, results from targeting other modifiable risk factors such as HTN and dyslipidemia have been mixed. Nevertheless, research has shown that even a decrease of about 10% in hemoglobin A1c (HbA1c) levels can reduce the risk of DR by as much as 39%. This is encouraging, as many interventions are now showing effectiveness even in the early stages of non-proliferative DR, where the retinal blood vessels begin to leak but the abnormal blood vessels characteristic of the later stage of proliferative DR have not yet begun [25].

Emerging literature highlights additional processes such as diabetic retinal neurodegeneration, microglial/glial activation, oxidative stress-mitochondrial dysfunction, and epigenetic regulation as contributors to early retinal dysfunction, often preceding overt microvascular pathology [26]. Integrating these processes into existing angiogenic models strengthens conceptual continuity across disease stages. Advances in molecular profiling have also drawn attention to non-coding RNAs, including microRNAs and long non-coding RNAs, as modulators of inflammatory and angiogenic signaling in diabetic retinal tissue [27], suggesting additional layers of regulatory complexity.

Although these areas are rapidly evolving and highly relevant, they were not explored in depth in the present review in order to maintain a focused narrative centered on hypoxia-driven angiogenic and inflammatory pathways. Future integrative reviews may benefit from synthesizing these emerging mechanisms with established vascular models to further refine disease classification and therapeutic targeting.

In parallel, recent clinical trials and real-world literature on newer agents and longer-interval regimens (including treat-and-extend strategies) are reshaping the therapeutic landscape of DR and DME [28], underscoring the need for continued alignment between molecular insights and evolving clinical practice.

Together, these findings underscore the importance of early diagnosis and timely intervention in improving outcomes and aiding in the prevention or slowing of this debilitating chronic disease.

Statistical Considerations

No meta-analysis, meta-regression, or formal statistical summary methods were planned or performed. Given the narrative scope and heterogeneity of included studies, additional statistical peer review was not required.

## Conclusions

This narrative review integrates foundational and recent molecular insights into the pathogenesis of DR. By clearly defining its narrative scope and mechanistic focus, the review aims to support clinicians and researchers seeking an accessible yet rigorous synthesis of hypoxia-driven retinal disease pathways. Newer research tools have opened the door to our understanding of the underlying cellular processes involved, though much of the molecular pathophysiology remains only partly understood. As therapeutic options grow, questions about the durability, affordability, and accessibility of these treatments will continue to linger. Care for diabetes itself is evolving. Newer medications, such as glucagon-like peptide-1 (GLP-1) receptor agonists, may ultimately reshape how diabetic eye disease develops over time. This makes it imperative to align emerging scientific advances with real-world clinical practice, not just to manage disease, but to slow its progression and prevent the most devastating outcome of all: permanent vision loss.
